# King’s College Hospital

**Published:** 1853-11

**Authors:** 


From the London Lancet.

### KING'S COLLEGE HOSPITAL.

*Tic Douloureux; Great Relief by Medical Treatment and Sub cutaneous Sections of the Nerve repeated at several months' interval; Relapse; Third Operation, including Section and Caulterization; Mitigation of Symptoms.*

(Under the care of Dr. TODD and Mr. FERGUSSON.)

The following passage in the preface to Malgaigne's "Operative Surgery" chimes so completely with the views which have always been entertained in this journal, that we take the liberty of quoting it in full :—

"A book on 'Operative Surgery' should, in order to satisfy the wants of the present race of surgeons, discuss for each operation—first, the indications and the surgical anatomy; pass in review all the methods which have been proposed; and, after a careful examination of the same, make a choice among them, assigning the reasons of the preference; and give a description of the different steps of the operative proceedings. The various modes of dressing the parts operated upon should then be pointed out; statistics of favorable and unfavorable results be adduced; the causes which led to the death of patients inquired into, by means of post-mortem examinations; and the measures best calculated to prevent a fatal issue carefully indicated. I have since been led to believe that even these details were not sufficient, for we are every day taught by experience nct to consider as perfectly cured those who apparently recover, and that it is very important to keep an accurate account of relapses, both as regards the nature of the affections and the operative methods which were adopted. Nor is this all, for it is extremely interesting, even after the most undoubted cures, to study and observe the consequences of each operation upon the organs, the functions, and the general condition of the patient. The relation of any case which does not include the latter information may be looked upon as incomplete, and considerations of this kind open a new and wide field to the exertions of our modern surgeons."

We were forcibly reminded of the preceding observations when we saw Mr. Fergusson's patient re-appear for the third time in the operating theatre. The recollection of several other cases, in which the eventual result had fallen short of the expectations formed at first, naturally came before us, and we became more convinced of the truth of M. Malgaigne's remarks. But it must be confessed that the present case belongs especially to the class of those in which relapses may be expected; indeed, the section of the nerve in tic douloureux has been often found to fail, and we cannot help suspecting that the cases of M. Jules Bonx, which we quoted in our last account of Mr. Fergusson's operation (THE LANCET, vol. ii. 1852, p. 316,) may, though at first successful, have shared the fate of the great majority of examples of tic, which, as is well known, are extremely difficult of cure.

It would nevertheless appear that the section and removal of about six lines of the inferior dental nerve at its entrance into the bony canal, has really and truly led to a complete cure in a case of Dr. Warren; even the simple subcutaneous section has succeeded in M. Bonnet's hands (this gentleman being the first who proposed to operate subcutaneously,) as also in a case mentioned by Mr. Fergusson after operating in the first place upon the present patient. It would, therefore, be well, supposing further attempts to be made, if the following rules, laid down by M. Malgaigne, were to obtain attention:—

1. The trunk of the affected nerve should be laid bare above the origin of all the branches in which pain is felt. 2. The parts operated upon should be relaxed so that no pulling of the nerves may be necessary. 3. The operator should ascertain, by raising and irritating the nerve, that he has really reached the seat of pain. 4. The nerve should be divided by a single cut, and as high up towards its origin as the wound will allow; a portion of the lower end of the nerve may then be removed without giving the patient any pain. 5. The portion excised should be as long as possible, and at least four or six lines should be taken away.

But it is well known that the two ends of a nerve, from which a portion *one inch long* was removed, have been found re-united (Swan); hence cauterization of the lower end of the divided trunk has been advised and performed; but relapses have occurred, notwithstanding this precaution. M. Malgaigne thinks that the reunion of the two ends might perhaps be prevented by isolating either one or both extremities of the divided nerve, and turning them round, so as to leave a kind of loop, after they had been soldered to the soft parts by the process of cicatrization; or else to detach a small piece of muscle from the surrounding soft parts, and interpose it between the two ends of the nerve; thus taking advantage of a pathological occurrence, which sometimes prevents union between the fragments of a broken bone.

It will be seen by the following particulars, that Mr. Fergusson adhered to most of the rules which have been thought best calculated to ensure success; the affection has, nevertheless, after three operations, been merely mitigated, and this fact will naturally appear of some weight to those who are not inclined to advise the section of the nerve. But the pain of tic douloureux is so distressing, and the ordinary means of relief are so powerless, that discouragement should not be allowed to paralyse further attempts, and some way will perhaps be found to act upon the nerve (be it the facial, infra-orbital, frontal, or dental) much higher up than has hitherto been attempted.

By referring to the number of this journal to which we have above alluded, it will be seen that, after giving a description of the subcutaneous section performed by Mr. Fergusson, we concluded by the following words:—"The patient progressed extremely well: the wound healed rapidly, and he left the hospital a few weeks after admission, freed, at least for a time, from the very severe pain to which he had so long been subject."

We now continue the account, aided by the notes of Mr. Plowman, one of Dr. Todd's clinical clerks. It would thus appear that the patient (who is a bricklayer, about forty years of age) left the hospital at the beginning of August, 1852, about one month after the first subcutaneous section of the mental branch of the inferior dental nerve. About eight or nine days after his discharge, the patient was again attacked by the old pain, which proved as severe as ever; he became an out-patient of the hospital, and was much relieved by a course of carbonate of iron. Sensation returned in the integuments of the lower jaw about five months after the division of the nerve, but the neuralgia, as stated above, had reappeared about five weeks after the operation, and was then controlled by the admission of carbonate of iron. The patient remained free from pain until the end of January, 1853, but it then attacked him again, and he was re-admitted on the 3d of February, about seven months after the first operation. He attributed this relapse to a cold, and the paroxysms of pain went on increasing in intensity till they were quite as severe as at any other period. The poor man complained of great flatulence, and a feeling of distension, with some dull pain in the stomach not increased by food; the bowels were open, but the evacuations had been much indurated.

The neuralgia is situated over the right half of the lower jaw; the patient describes it as like the passing of red-hot wires through the cheek, and says it appears to start from the front of the ear, its chief severity being felt about the angle of the mouth; and towards the end of each attack it seems, he says, to radiate directly upwards from the same spot to the top of the head. It occurs in very frequent paroxysms (about every five minutes), and is very liable to be induced by movements of the jaws or lips, as by talking, &c. Violent friction appears to numb the pain, and is the only way in which he can at all relieve it.

After a warm bath and an alkaline purge, he was ordered five grains of carbonate of iron to be taken three times a day; but his digestive organs became deranged, and the bowels obstinately confined. The usual treatment was had recourse to for these symptoms, and about three weeks after admission the carbonate of iron, in ten-grain doses three times a day, was resumed. The neuralgia began to abate. Dr. Todd ordered subsequently iodide of potassium; finally oil of turpentine, and opium, and on the 8th of March, about two months after admission, the paroxysms were much less in severity; the patient suffered only two or three times a day, slept well, and was discharged, considerably relieved, about one month after the second admission.

The paroxysms of pain suddenly returned one month after the patient's discharge, and recurred every quarter of an hour. He was re-admitted May 5th, 1853, at which period the paroxysm had become very frequent, recurring every three or four minutes. Dr. Todd ordered a warm bath, middle diet, and three minims of Fowler's solution in an ounce and a half of water, to be taken three times a day after meals. The pain was, however, not diminished by these means; it spread, on the contrary, to the whole of the right side of the face, and was no longer confined to any particular spot.

Thirty minims of chloroform were now inhaled four times a day, and soon afterwards, tincture of aconite was also rubbed in where-ever there was pain, the bowels were at the same time kept quite free by the administration of scammony and colocynth every night. The paroxysms remained just as frequent as before, but diminished in intensity; and in a few days the purgative pills were changed to ten grains of colocynth, to which was added the sixth of a drop of croton oil; twenty minims of Brandish's potash-water were also given three times a day.

The improvement was very slight, and Dr. Todd considered that the time had now come to try the narcotic effects of tobacco. The patient was therefore desired to smoke every morning two drachms of that preparation of the leaf known under the name of Cavendish. The tobacco had not the desired effect, nor did it agree with the digestive organs; and the patient expressed, under these circumstances, the wish of having the nerve again divided by Mr. Fergusson. A second subcutaneous section was therefore undertaken on the 20th of May, 1853; it proved availing for a time, but the pain returned a few weeks after the patient had left, and he was admitted for the third time in August, 1853.

On the 20th he was brought into the theatre, and having been narcotized by chloroform, Mr. Fergusson made a semi-circular incision over the spot where the dental branch of the inferior maxillary nerve emerges from the bone. After some dissection, a greyish mass, looking like a neuromatous concretion, was brought into view; this was removed, being looked upon by Mr. Fergusson as the result of former operations. Nor is it at all unlikely that nervous bulbs were generated after the two preceding subcutaneous sections, and this would go far to make us look upon these sections (though advocated by such a good surgeon as M.

Bonnet, of Lyons) as extremely likely to aggravate the disease. This irregular state of parts completely obscured the relative anatomy of the region, and no distinct longitudinal piece of nerve could be cut away; but Mr. Fergusson used the actual cautery in the direction of the mental foramen, both to arrest hæmorrhage and to destroy as much as possible of the nerve within the canal. The flap made to bring the parts into view was then laid down again, fixed in the usual way, and the patient removed.

Mr. Fergusson remarked, after the operation, that he had now divided the mental branch of the inferior maxillary nerve for the third time upon the same patient; the two first times he had merely cut the nerve across, by means of a sub-cutaneous incision, and on each occasion considerable relief had been obtained, but he had now resolved to lay bare the nerve, and cut off a portion of it.—The neuromatous mass, which was seen during the dissection, was probably the consequence of the former operations, and it was to be hoped, now that the two ends of the nerve were so widely separated, and the trunk had been cauterized within the canal, that the benefit would be somewhat more lasting than it had hitherto been. Proceedings of this kind had long been in abeyance, but as the patient felt no pain when under the influence of chloroform, there could be no objection to resort to the section of the nerve, as a *dernier ressort* in cases of facial neuralgia.

We carefully watched this patient from the date of the operation (August 20) to September 22, when he was discharged; and it was evident that the operation had considerably relieved him. The wound was, at the time of his leaving the hospital, quite cicatrized, and scarcely visible, and on being questioned, the man stated that the pain was not by far so severe as it had been, but there remained a gnawing sensation which annoyed him very much.

We think it unnecessary to add any comments to the relation of this case; if the patient has now less pain, the operation (especially with the aid of chloroform) seems to us very justifiable, and we would merely call attention to the gradual spread of the nervous inflammation which must have taken place since the disease begun. Originally the pain was confined to the base of the jaw in the vicinity of the chin; but on the third admission it is described as "starting from the front of the ear, the pain being chiefly feft at the angle of the mouth, and towards the end of the attack the distressing sensation seemed to radiate from the same spot to the top of the head." Now does this imply that the facial, infra-orbital, or frontal branch of the supra-orbital nerves, became implicated? As to the facial, many pathologists (and among them M. Berard) have denied that it could be the seat of neuralgia; but, however that may be, it is plain that the affection known under the name of facial neuralgia, is apt to spread and implicate several other nervous branches and trunks; and this is a sufficient reason for not allowing the disease to take its course, and should be an incentive for surgeons to try every means of checking the progress of the affection.

When alluding to this case, now just twelve months ago, we stated that tic was rarely met with in our hospitals: we have no reason to modify this opinion, for we have not seen *one case* in our rather extensive nosocomial circuit since last we mentioned the disease. One remark we would finally make respecting the spot where the nerve, on further trials, might advantageously be attacked, and on the manner of cauterizing the ends of the nervous trunk. Though the use of trepine seems somewhat severe, it would, perhaps, be useful to bring the dental branch to view just as it is entering the canal, by raising a flap over the proper region, and using the trephine. If it were desired to cauterize, besides taking away a piece of the nerve, the platinum wire might conveniently be introduced cold, either into the bony canal, or between the pyterygoid muscles, and when in proper position made red-hot by means of the battery, according to the plan introduced by Mr. Marshall, of University College Hospital, and lately put forth at Paris, by the son of M. Amussat, as an *original* contrivance.

In a medical point of view we should not overlook the good results obtained in this case by the carbonate of iron, which acted so beneficially in Dr. Todd's hands. This circumstance should encourage us to persevere longer than is generally done in the use of martial preparations; the same being aided by all those hygienic and dietetic means which are known to invigorate the system.

